# Cerebellar-Motor Dysfunction in Schizophrenia and Psychosis-Risk: The Importance of Regional Cerebellar Analysis Approaches

**DOI:** 10.3389/fpsyt.2014.00160

**Published:** 2014-11-25

**Authors:** Jessica A. Bernard, Vijay A. Mittal

**Affiliations:** ^1^Department of Psychology and Neuroscience, University of Colorado Boulder, Boulder, CO, USA; ^2^Center for Neuroscience, University of Colorado Boulder, Boulder, CO, USA

**Keywords:** cerebellum, schizophrenia, psychosis-risk, motor abnormalities, balance, timing, morphology, motor learning

## Abstract

Motor abnormalities in individuals with schizophrenia and those at-risk for psychosis are well documented. An accumulating body of work has also highlighted motor abnormalities related to cerebellar dysfunction in schizophrenia including eye-blink conditioning, timing, postural control, and motor learning. We have also recently found evidence for motor dysfunction in individuals at ultra high-risk for psychosis (1–3). This is particularly relevant as the cerebellum is thought to be central to the cognitive dysmetria model of schizophrenia, and these overt motor signs may point to more general cerebellar dysfunction in the etiology of psychotic disorders. While studies have provided evidence indicative of motor cerebellar dysfunction in at-risk populations and in schizophrenia, findings with respect to the cerebellum have been mixed. One factor potentially contributing to these mixed results is the whole-structure approach taken when investigating the cerebellum. In non-human primates, there are distinct closed-loop circuits between the cerebellum, thalamus, and brain with motor and non-motor cortical regions. Recent human neuroimaging has supported this finding and indicates that there is a cerebellar functional topography (4), and this information is being missed with whole-structure approaches. Here, we review cerebellar-motor dysfunction in individuals with schizophrenia and those at-risk for psychosis. We also discuss cerebellar abnormalities in psychosis, and the cerebellar functional topography. Because of the segregated functional regions of the cerebellum, we propose that it is important to look at the structure regionally in order to better understand its role in motor dysfunction in these populations. This is analogous to approaches taken with the basal ganglia, where each region is considered separately. Such an approach is necessary to better understand cerebellar pathophysiology on a macro-structural level with respect to the pathogenesis of psychosis.

## Introduction

Schizophrenia is a devastating mental illness marked by a variety of symptoms, including positive symptoms (hallucinations and delusions) and negative symptoms (anhedonia and social withdrawal) (5). In addition to the classic symptoms, these patients also exhibit movement abnormalities as well as cognitive and affective dysfunction. These movement abnormalities are in a variety of domains and include dyskinesias (6), as well as psychomotor slowing, catatonia, neurological soft signs, extrapyramidal signs, and motor learning deficits (7–10). Psychosis has been studied as a spectrum disorder, with investigations of different patient groups that show some symptoms that are associated with risk. In this review, we focus on several specific groups in addition to schizophrenia, including those at psychosis-risk, a category that can refer to genetic risk, as those with a first-degree relative with the disease are at greater risk for development of schizophrenia. In this category, there are also individuals referred to as ultra high-risk (UHR), where there are recent onset or escalating range of moderate (i.e., partially formed/occurring occasionally without full conviction or related functional impairment) positive attenuated symptoms (e.g., unusual thoughts, suspiciousness, grandiosity, perceptual anomalies, and disorganized communication) associated with decreased functionality in social relationships or day-to-day life. After a diagnosis of schizophrenia, this period of attenuated symptoms is referred to as the prodromal period. However, not all those that experience these symptoms will go on to develop schizophrenia or another psychotic disorder. Finally, there are individuals diagnosed with schizotypal personality disorder, who show trait level unusual behaviors, suspiciousness, and ideas of reference. This group is also at greater than average risk for schizophrenia. Interestingly, movement abnormalities are present in these at-risk populations as well [e.g., (1, 2, 6)].

Movement abnormalities are present as early as infancy in individuals that go on to develop schizophrenia later in life [e.g., (5)], indicating that such movement abnormalities maybe associated with the disease or disease process, as opposed to being a side-effect of medication (11). Indeed, prior to the onset of schizophrenia, and certainly during infancy, individuals are less likely to be impacted by many of the confounding factors that could also further impact movement abnormalities associated with schizophrenia (e.g., anti-psychotic medications, drug, and alcohol use/abuse). These movement abnormalities are typically dyskinetic movements, and are thought to be related to the dopaminergic dysfunction in the basal ganglia (12), and have been previously reviewed elsewhere (6). Importantly, these motor abnormalities are present prior to disease onset, and continue across the disease course. Further supporting this, many movement abnormalities are seen in drug-naïve adults with schizophrenia as well (10).

In addition to the striatally mediated dyskinesias seen in schizophrenia and psychosis-risk groups (3, 6, 13–22) and the psychomotor slowing, catatonia, and parkinsonism (7–10), there is also evidence of cerebellar dysfunction in both schizophrenia, and psychosis-risk populations [e.g., Ref. (1–3, 17–21)]. The most prominent evidence of cerebellar dysfunction is with respect to its role in cognitive dysmetria (23–25). Andreasen and colleagues have demonstrated that there is abnormal functional activation in the cerebellum, thalamus, and cortex in patients with schizophrenia (23, 24), and more recently, there has been increased interest in the study of the cerebellum in these patients, particularly with respect to cerebello-thalamic connections (26). Cerebellar activation deficits exists across functional domains (27), and we refer readers to the review by Picard and colleagues for a detailed discussion of the cerebellum in schizophrenia, as it is beyond the scope of our review here (25).

The cerebellum is classically thought of as a brain structure that is important for motor control; however, there is a robust literature to indicate that the structure is also heavily involved in cognitive and affective processing (4, 28–33). As such, though the cerebellum and cerebello-thalamo-cortical circuit were first discussed in schizophrenia with respect to cognitive dysmetria, there is also a great deal of evidence to indicate that there are motor cerebellar deficits in schizophrenia in addition to the aforementioned dyskinesias, psychomotor slowing, catatonia, and parkinsonism. Indeed, there is also an emerging literature indicating that as with dyskinesias, cerebellar-motor abnormalities are also present in psychosis-risk populations [e.g., (1, 2)]. Thus, it may be that more overt cerebellar-motor dysfunction may also serve as a marker of disease and disease progression, such as has been proposed for striatal-mediated dyskinesias (6). This is particularly exciting, as the cerebellar–thalamic network represents a distinct mechanism, and may hold the potential to explain/predict heretofore poorly understood processes across the psychosis spectrum. Here, our goal is twofold. First, we provide a review of cerebellar–motor dysfunction in patients with schizophrenia as well as in psychosis-risk groups including genetic risk, UHR populations, and schizotypal individuals. Second, we provide a detailed overview of the cerebellar functional topography and weigh evidence in support of a targeted regional approach for cerebellar investigations. We suggest that more specific topographically informed approaches to investigating the cerebellum (particularly with respect to cerebellar morphology and cerebello-cortical networks) across the psychosis spectrum will yield informative results with respect to the involvement of the cerebellum in psychosis.

## Motor Cerebellar Dysfunction in Schizophrenia and Psychosis-Risk

The cerebellum is important for motor function generally in that it is important for online monitoring of movements (34, 35). Eye-blink conditioning and postural control are both especially reliant upon the cerebellum, but it is also strongly engaged during motor learning, and it has been suggested that one of the primary functions of the cerebellum is in the precise timing of movements (36, 37). While the cerebellum is likely to be involved in many motor behaviors, several domains have been studied in greater depth in patients across the psychosis spectrum. We will discuss in turn eye-blink conditioning, postural control, timing, and motor learning. For each motor task paradigm, we will review the existing evidence indicating impairments across the psychosis spectrum, as well as any evidence that directly links these behaviors to the cerebellum [either structure or function as measured using functional magnetic resonance imaging (fMRI)] in these populations. A summary of performance for each task domain for both patients with schizophrenia and at-risk/psychosis spectrum populations is provided in Table 1. Finally, future directions for research in each domain will be discussed, particularly with respect to the study of psychosis.

**Table 1:** **Summary of findings across the reviewed motor domains for both patients with schizophrenia and high-risk groups**.

Task domain	Schizophrenia deficits	High-risk deficits
Eye-blink conditioning	Poor conditioning: fewer conditioned responses to the presentation of a tone, which is followed by an aversive puff of air to the eye	Fewer and earlier conditioned responses in SPD
	Altered timing of responses so that eye does not close in time with the air-puff	Genetic risk populations also show fewer conditioned responses
Postural control	Greater postural sway indicative of poor postural control, associated with symptom severity	Increased postural sway in UHR individuals that is specifically associated with negative symptom severity
	Presence of the Romberg sign more common in patients	
Timing	Impairments in temporal bisection (time perception)	No evidence indicating sub-second cerebellar timing deficits
	Increased variability during temporal production (synchronization–continuation tasks)	Sub-second timing correlated with dimensions of schizotypy
Motor learning	Implicit and explicit sequence learning deficits such that patients learn, but to a lesser degree than controls	Deficits in pursuit rotor performance in UHR
	Relationships also seen with the cerebellum	No deficits on sequence learning in SPD

*SPD, schizotypal personality disorder; UHR, ultra high-risk*.

### Eye-blink conditioning

Eye-blink conditioning is one of the simplest learning paradigms, and performance of this task is dependent upon the cerebellum (38–40). The circuits involved in eye-blink conditioning have been carefully mapped using animal models, but have also been extensively investigated in humans primarily in lesion studies, and both the deep cerebellar nuclei (particularly the interposed nuclei) and cerebellar cortex seemed to be important for task performance (38–40). In these paradigms, a puff of air is delivered to the eye, resulting in a blink response. During the conditioning phases, a tone precedes and typically co-terminates with the air-puff, resulting in conditioned responses (CRs). That is, individuals that learn the association between the tone and the air-puff that follows it blink their eyes at the sound of the tone. Because this is so heavily dependent upon the cerebellum, it is often used as an indicator of cerebellar function. In schizophrenia, there is a growing literature investigating performance on this task in patients.

Across the literature investigating patients with schizophrenia, the general finding is that patients are impaired in eye-blink conditioning, and show fewer CRs to the tone alone when compared with controls (40–47). During the paradigm, the percentage of CRs is significantly lower than that in controls. However, though this seems to be a relatively robust finding, there have been several studies that provide evidence to the contrary (48–51). Reasons for the mixed findings are unclear, but may be due to the heterogeneity of the patients included in these samples particularly with respect to medication, as well as differences in task design. Importantly, a recent investigation of neuroleptic naïve patients or patients that have been off medication for 3 weeks demonstrated eye-blink conditioning deficits suggesting that this impairment is not solely a function of medication status (46).

In addition to the lower percentage of CRs, there is also evidence in patients with schizophrenia to indicate that the timing of these responses is negatively impacted (41, 42, 45) (timing will also be discussed in further detail below). During the conditioning paradigms, the tone typically co-terminates with the onset of the air-puff to the eye. The optimal response is such that the anticipatory eye-blink that occurs as a result of the tone (CR) is timed so that the eye is closed upon the delivery of the air-puff. Brown and colleagues found that the timing of the CRs was more variable in patients with schizophrenia (42) while Bolbecker and colleagues reported less adaptive CRs as they occurred significantly earlier in patients when compared to controls (41). A similar trend level finding of earlier CRs in patients was found by Forsyth and colleagues (45).

Across the psychosis spectrum, there is also evidence to indicate impairments in eye-blink conditioning. Individuals with schizotypal personality disorder have deficits similar to patients with schizophrenia in that they show fewer CRs, and there is also a trend indicating altered (earlier) CR timing in this population (45). Most recently, Bolbecker and colleagues investigated eye-blink conditioning in a group of patients with schizophrenia, those at genetic risk, and healthy controls (52). Both the patient and genetic risk groups showed impaired associative learning as measured with CRs. The authors suggest that cerebellar abnormalities may be a marker of risk for schizophrenia (52). Furthermore, this provides additional support to indicate that these deficits are not an artifact of medication, as they are present in genetic risk groups.

Finally, it is of note that there have been several investigations of the cerebellum more directly with respect to eye-blink conditioning. First, Edwards and colleagues measured the volume of the anterior and posterior aspects of the cerebellum and investigated these volumes with respect to conditioning (43) in patients with schizophrenia and controls. Volume of the anterior cerebellum in patients was smaller than that of controls and the patients also showed impaired conditioning. However, there were no significant correlations between cerebellar volume and conditioning performance in patients with schizophrenia. In fact, the non-significant relationship in the patient group was in the opposite direction as that seen in the controls (43). The authors suggest that there are perhaps altered structure–function relationships in the cerebellum in patients with schizophrenia (43). However, further confirmation of these findings is necessary, particularly as only very large regions of the cerebellum were investigated.

Using positron emission tomography (PET), medication free patients with schizophrenia and controls were scanned during the eye-blink conditioning paradigm (46). Not only did the patient group show impaired associative learning, but they also showed decreased blood flow in the cerebellum and the thalamus. This indicates cerebellar dysfunction in the patient group along with the behavioral impairments (46). While this investigation and that of Edwards and colleagues are important first steps in linking cerebellar morphology and function to this overt motor impairment, it is also clear that more work is needed.

There are several key future directions for eye-blink-conditioning research. Most interestingly is the use of functional neuroimaging. While Parker and colleagues took advantage of PET imaging (46), recent advances in the technology used to deliver the air-puff and monitor the eye-blinks have allowed for investigations of this task using fMRI (53, 54). This approach has provided important insights into the cerebellum and eye-blink conditioning in normative development (54). Linking eye-blink conditioning to additional cognitive measures as well as symptomatology in disease populations is important. Several groups have started to investigate eye-blink conditioning with respect to cognition with mixed results (41, 45), and to our knowledge there have not yet been any investigations linking CRs with symptom severity. Such future work is important for our understanding of cerebellar contributions to disease and cognition, and will help us to better understand whether or not this motor measure is a reasonable marker of disease state. Lastly, investigations in UHR populations would also be especially informative. The work by Bolbecker and colleagues in genetic risk is an important first step (52), and the field would benefit from a replication and extension of this work in UHR individuals.

### Postural control

Postural control relies upon sensorimotor integration and vestibular function. The cerebellum has long been implicated in postural control (55). Patient studies in those with ataxia or cerebellar lesions show increased postural sway (poor postural control) (56), and the measurement of regional cerebral blood flow using PET imaging has also shown increases in blood flow in the cerebellum (57). Much like eye-blink conditioning, postural control can also be used as a potential indicator of cerebellar function. Indeed in healthy adults, balance has been linked to regional cerebellar volume (58), and similar associations between cerebellar volume and balance have been seen in alcoholism where cerebellar volume is negatively effected by the disease (59, 60).

Investigations of postural control in schizophrenia were initiated as far back as the 1940s. Using vestibular stimulation Angyl and Sherman (61) found deficits related to postural control in patients with schizophrenia. Though the methods to investigate postural control vary greatly in their sensitivity, the general finding is that patients with schizophrenia have impaired postural control. Earlier work in this domain relied primarily upon behavioral measures and assessments of balance such as judgment of the presence of the Romberg sign (loss of balance when the eyes are closed, arms are outstretched, and feet are in a heel-to-toe tandem position), or assessing the ability to stand heel-to-toe, or on one foot (62, 63). Presence of the Romberg sign is significantly more common in patients with schizophrenia as compared to healthy controls (62) and patients are also impaired at standing on one foot, and heel-to-toe standing, though this is further compounded in patients with schizophrenia that also have a history of alcoholism (63). Thus, in schizophrenia, these postural control deficits are present, and can increase in severity in cases of alcohol abuse.

More recent investigations have used instrumental measures of balance and quantify body sway. Such measures have a much higher degree of sensitivity in their ability to detect postural abnormalities. Postural sway is quantified, and a greater degree of sway is indicative of poorer postural control. Furthermore, these instrumental measures also allow researchers to manipulate the placement of the feet, and whether or not participants complete the task with their eyes opened or closed. Marvel and colleagues (64) were the first to use such a measure of balance to investigate postural control in patients with schizophrenia. This investigation demonstrated that patients with schizophrenia have deficits in postural control such that they sway more than controls, though they did not see any further effects of alcohol use (64). These findings were recently replicated by Kent and colleagues (65), and they demonstrated that in patients, greater postural sway was associated with worse general psychopathology symptoms. There was a similar trend with respect to negative symptoms (such as anhedonia). Importantly, in both of these investigations anti-psychotic medications do not seem to be impacting the findings (64, 65). The findings of increased sway were also replicated across a heterogeneous group of patients with psychosis, including those with schizophrenia, acute psychosis, and undefined psychotic disorder (66). It is of note, however, that an additional recent study is not consistent with these findings (67).

To our knowledge, there has been only one study investigating postural control in UHR individuals. We recently investigated whether or not adolescents and young adults at UHR for psychosis show impaired postural control as measured by increased sway area (1). We found that postural control deficits are indeed present in UHR individuals. Furthermore, greater sway area was associated with increased negative symptoms. Finally, we investigated cerebello-cortical networks using resting state connectivity MRI. Not only were cerebello-cortical networks weaker in the UHR group relative to the controls, but they were also correlated with postural sway, providing a link between cerebellar networks and behavior in this population (1).

Future directions regarding postural control across the psychosis spectrum fall into several key domains. First is the use of instrumental measures to quantify postural sway. Since Marvel and colleagues first used this method in patients with schizophrenia (64) there have been several replications. Using such methods whenever possible provides a more sensitive measure of postural control, and also allows for better comparison across investigations. Relatedly, such methods also lend themselves to more complex statistical analysis techniques, which may yield additional important information regarding postural control and schizophrenia, as demonstrated by Kent and colleagues (65). Second, additional investigations and replications of our recent findings regarding postural control deficits in UHR populations (1) are warranted. Follow-ups across disease progression in longitudinal investigations will also be especially informative. Finally, more direct links with cerebellar structure and function in patient groups are needed. While there is strong evidence in healthy individuals and in other clinical populations linking the cerebellum more directly to cerebellar structure and function (57, 58, 63), such work is lacking across the psychosis spectrum.

### Timing

The cerebellum has been implicated in timing function across multiple research domains. Assessments in cerebellar patients have indicated that these individuals are impaired in both timing production and perception (68), and more recently, these impairments have been linked more specifically to discontinuous timing tasks, such as discrete finger tapping (69). Furthermore, functional neuroimaging methods have also implicated the cerebellum in timing perception (70, 71). Overall, the cerebellum is thought to be generally very important in timing, particularly with respect to precise event timing (72). It has been suggested that the cerebellum is particularly important in timing on the sub-second scale, and that longer timing intervals (supra-second) are more cognitively mediated, and may be related to the basal ganglia (72). On the whole the cerebellum is certainly implicated in timing and this is important for a wide array of motor tasks such as finger tapping and sequence learning. With respect to schizophrenia, there is evidence to indicate that timing functions, particularly those that are purported to be cerebellar-dependent are impaired. Here, we will focus primarily on sub-second timing, as this is most closely linked to the cerebellum.

Before discussing the timing deficits seen in patients with schizophrenia, it is important to understand the more common methods to investigate timing. Broadly speaking, these paradigms fall into two categories – time perception and production. The most typical way of assessing time perception is with a temporal bisection task. During a temporal bisection task, participants are presented with anchor durations that are either long or short. Then, test durations are presented and participants are asked to determine if the test duration is closer to the long or short anchor, and timing variability and temporal precision can be quantified [e.g., (48)]. Production tasks typically involved finger tapping using a synchronization–continuation type paradigm. Participants synchronize their tapping to a tone, and the tone is then taken away while tapping continues. Tap variability, often measured as the coefficient of variation, is used to quantify timing in these paradigms [e.g., (49)]. In both cases, the intervals used can vary to include both sub- and supra-second timing.

While investigations of timing in schizophrenia are certainly nothing new, earlier work largely focused on intervals of several seconds [e.g., Ref. (50–52)], which are thought to be more cognitively demanding, and are less likely to involve the cerebellum. More recent work, however, has investigated these sub-second durations in schizophrenia. The first of these investigations was by Elvevåg and colleagues (73). This study included two tasks, a temporal bisection task, as well as a temporal generalization task where participants had to recognize a standard duration. Across both domains, patients with schizophrenia were impaired with respect to controls, and importantly, performance was not correlated with working memory abilities, nor was it strongly associated with general intelligence (73). Davalos and colleagues replicated these findings even when the time between the anchor and test durations was varied (74), and similar results are seen with stimuli in both the auditory and visual domains (75). The patient group was impaired in both domains, but the impairment was greatest for the auditory presentation of stimuli. Interestingly, deficits have also been seen in patients with schizophrenia that have first-rank symptoms (76). These individuals experience hallucinations and thoughts that they believe to be under the control of another agent. The authors suggest that these patients may have a slowed internal pacemaker such that they experience time differently. However, it is crucial to note that these differences were not present in patients without first-rank symptoms (76), though it is possible that such sub-groups may be driving the effects in other investigations.

As noted above, sub-second intervals are thought to be more reliant upon the cerebellum, while supra-second intervals rely upon other neural systems, perhaps the basal ganglia, and are postulated to be more cognitively demanding (72). While the primary focus here is on cerebellar-mediated motor behaviors, work by Carroll and colleagues comparing temporal bisection performance on sub- and supra-second durations is worth noting (77). In this investigation, the patients were impaired in both timing ranges and the authors suggest that this may be indicative of a more general timing deficit in schizophrenia (77). There were also no associations with time deficits and symptomatology. However, it is worth noting that the sub-second findings not only support the cerebellar deficits in schizophrenia, the basal ganglia, and pre-frontal cortex, which are important for more cognitively mediated tasks, have long since been implicated in schizophrenia (78). Thus, the longer supra-second durations may be tapping into additional neural systems that are impacted by the disease.

The majority of timing work has been done using time perception. In the one study, we know of using a synchronization– continuation time production task, there are also deficits in patients with schizophrenia as compared to controls during both the synchronization and continuation phases (79). During this task, tapping variability was increased in patients during both task phases. Furthermore, models of timing indicated that in patients with schizophrenia the deficit was due to actual deficits in timing as opposed to task performance or implementation (79). The motor production aspects of timing production may also come into play in motor learning tasks that involve timed, sequential finger movements (please see below).

Across the psychosis spectrum and in UHR individuals there has been very little work to date on timing. In those at genetic risk, a timing deficit was found, but this study was limited to supra-second durations, and similar supra-second deficits are seen in those with high schizotypy based on the Schizotypal Personality Questionnaire (80, 81). Thus, investigations of sub-second cerebellar-mediated timing tasks are necessary in UHR populations. However, sub- and supra-second performance was correlated with schizotypy dimensions (81). Overall, it is clear that further work is needed in these populations to better understand cerebellar-mediated timing deficits.

Future directions with respect to timing deficits fall into several domains. Most importantly is the need for additional work using temporal production tasks at the sub-second level in patients with schizophrenia. Relatedly, there is a general lack of research on this domain in at-risk populations and across the psychosis spectrum. Understanding whether or not such sub-second timing deficits exist prior to the onset of formal psychosis will provide us important insight as to the range of cerebellar-motor dysfunction prior to disease. Next, translation of such tasks to the scanner environment using functional neuroimaging is warranted, as are investigations looking at associations with regional cerebellar volume. While studies in individuals with cerebellar damage (69) and those using functional neuroimaging (70, 71) have implicated the cerebellum, such measures in schizophrenia and across the psychosis spectrum will further our understanding of the nature of this timing deficit in patients. Similarly, insightful relationships with regional cerebellar volume may be gleaned, and indeed Ivry and Spencer have suggested that there may be regional contributions of the cerebellum to timing (72). Finally, more work looking at timing with respect to symptom severity is needed.

### Motor learning

Motor learning is the process by which individuals learn to use new tools or devices, and turn novel and perhaps disjointed movements into fluid performance. Examples include learning to use a new computer mouse, or putting together a sequence of movements to shoot a basketball. The process of motor learning recruits a variety of cortical and subcortical brain regions (82–87), including the cerebellum. While it is certainly not the case that the cerebellum is engaged alone in motor learning, it does seem to be a key contributor, and investigating motor learning in patients with schizophrenia and those at UHR may be especially informative for our understanding of cerebellar–motor dysfunction in psychosis.

There are several different motor learning paradigms that are most typically used, and they seem to engage slightly different regions of the cerebellum (88). Motor sequence learning typically requires participants to learn a new sequence of finger movements, and both accuracy and reaction time are compared to a random sequence of button presses. This can be done explicitly where the participant knows they are learning a sequence, or implicitly when a sequence is learned while the participant is unaware. Mirror-drawing is a form of implicit motor learning that requires participants to update their movements based on a mirror-reflection of their movements. Over multiple trials, participants are able to accurately trace complex shapes. Pursuit rotor tasks, which are also implicit, ask participants to track a target across a track pad using a mouse or joystick. The target is titrated so that the target moves with varying speed in order to ensure a standard minimum level of accuracy and time on target is calculated. Over several trials, the time on target increases, indicative of learning.

Early work investigating motor learning in schizophrenia was behavioral in nature. Deficits on a pursuit rotor task were demonstrated by Schwartz and colleagues (89). Patients with schizophrenia spent less time on the target and deficits were exacerbated by advanced age. Furthermore, these findings were not associated with medication or other movement abnormalities in the patient group, though they were weakly related to cognitive abilities. Using an implicit sequence learning task (serial reaction time task), Green and colleagues investigated motor learning in patients with schizophrenia (90). While both patients with schizophrenia and healthy controls showed overall improvement in reaction time over the course of the task, the patient group showed less learning. The authors suggested that this may be due to possible cerebellar deficits (90). Looking at both implicit and explicit sequence learning, Pederesen and colleagues found deficits in both domains relative to controls in those with first-episode schizophrenia (91). Using mirror-drawing paradigms, patients with schizophrenia have been shown to have implicit learning adaptation deficits (92–94). However, these deficits are often linked to medications in these patients.

These initial behavioral findings were soon followed up by neuroimaging investigations using functional, anatomical, structural, and connectivity methods (95–100). Though the measures of motor learning varied to some degree, with one exception (100), the behavioral findings were consistent with prior work indicating deficits in motor learning in patients with schizophrenia. In addition, these neuroimaging investigations also provide further information about what underlying brain differences may be contributing to these deficits.

Kumari and colleagues (95) showed that there are differential brain activations when comparing patients to controls, and this included both the cerebellum and regions in the basal ganglia. The patients with schizophrenia did not activate these regions, though they were activated by controls (95). The implication of the cerebellum is perhaps not surprising, and interesting given the proposed role of the cerebellum in schizophrenia. Implicit sequence learning has also been investigated in patients with schizophrenia using PET (96). The patients showed less learning over time when compared to the control participants. In the patient group, the pre-frontal cortex and cerebellum showed differential correlations with sequence learning, highlighting the importance of the cerebellum, but also the importance of investigating the interactions between the cerebellum and pre-frontal cortex. Null findings with regards to learning and the cerebellum in schizophrenia have also been reported (100). Recently, using meta-analysis, we investigated cerebellar functional activation across a variety of task domains including motor function (27). The majority of included motor studies related to finger tapping and motor sequence learning. While we cannot speak to performance in our analyses, across these studies we did find that in patients with schizophrenia cerebellar functional activation was altered relative to controls during motor tasks, indicating that perhaps patients with schizophrenia rely upon less efficient cerebellar networks and processing (27).

As noted above with respect to the findings of Marvel and colleagues (96) investigating the interactions between the cerebellum and cortex is potentially of great interest. One way to do so is with functional connectivity analyses. These analyses measure the correlations between the brain signal in different brain regions. Recently, Kasparek and colleagues (97) investigated motor sequencing abnormalities with respect to functional connectivity between the cerebellum and cortex assessed while subjects were making finger movements (finger-to-thumb opposition). Motor sequencing was indexed based on the Neurological Evaluation Scale (NES). Both patients and controls were assessed and then divided into those with sequencing abnormalities and those without, regardless of diagnosis. However, motor sequencing deficits were more common in the patient group. There were no differences between patients with schizophrenia and controls with respect to functional connectivity; but, those with motor sequencing deficits had lower functional connectivity between the cerebellum and the motor cortex (101). Though the differences were not related to diagnosis, given that motor sequencing deficits were more common in the patient group, this finding is potentially important for understanding motor learning deficits in schizophrenia, and further highlights the role of the cerebellum.

Also relying upon the NES measure of sequencing, Hüttlova and colleagues recently looked at structural connectivity of the cerebellum using diffusion tensor imaging (DTI) (98). In the patients with motor sequencing deficits, there was decreased white matter structural integrity relative to controls in the superior cerebellar peduncle, which is the primary cerebellar efferent to the thalamus, whereas in the patients without deficits, differences relative to controls were seen in the corticospinal tract. This suggests potential sub-groups within schizophrenia related to motor sequencing, but also further highlights the cerebellum in motor deficits in this patient population.

Finally, structural MRI has been used with respect to sequence learning in patients with schizophrenia (99). Volumes of the cerebellum and pre-supplementary motor area (SMA) were investigated. First, the only group differences in volume were seen in the pre-SMA, and in patients volume in this region was correlated with implicit learning. While it is notable that there were no group differences in total cerebellar volume, nor were there any cerebellar-behavior relationships, the measurement of the entire structure may be a contributing factor. In sum, across multiple studies, there seem to be relatively robust deficits in motor sequence learning and in procedural learning in patients with schizophrenia, and this is supported by meta-analysis (102). Furthermore, there is at this point at least some evidence linking these deficits to the cerebellum, along with other liable neural substrates.

Though there has been a good deal of investigation related to motor learning in patients with schizophrenia, across the psychosis spectrum this has been investigated less extensively. In schizotypal individuals relative to controls, there do not appear to be any deficits in motor sequence learning, as measured using the implicit serial reaction time task (103, 104). However, more recently, we demonstrated procedural learning deficits in UHR patients relative to controls using a pursuit rotor task (2). Furthermore, learning was associated with volume of Crus I of the cerebellum. Given the cognitive functions of this region (4), and its associations with the pre-frontal cortex (105), this is an especially interesting finding, and may perhaps be related to the differing relationships with learning seen in the pre-frontal cortex and cerebellum (96). This may be due in part to the overall difficulty of this task as more complex motor tasks recruit this region of the cerebellum (106), though we may also be tapping into cognitive deficits as well.

Future directions in motor learning research across the psychosis spectrum include the further investigation of motor learning in psychosis spectrum populations. While there have been some inroads in this domain, further research is clearly warranted. Additionally, interesting investigations using non-invasive brain stimulation to the cerebellum have been completed in healthy individuals (107, 108). This stimulation can influence motor learning in these healthy individuals, and the impact on motor learning in patients with schizophrenia or across the psychosis spectrum may be especially informative. Finally, while there has been a great deal of work looking at the functional MRI correlates of motor learning in patients with schizophrenia, inclusion of anatomical and structural connectivity measures will be especially informative, both with respect to cerebellar pathology, but also to other brain regions implicated in motor learning.

Importantly, across all of these domains medication and cognitive deficits may be impacting performance. For example, deficits in mirror-drawing seem to be largely tied to medication (92–94), and our recent findings with respect to the cerebellum and pursuit rotor implicate cognitive cerebellar regions (2). Findings of cerebellar–motor deficits in at-risk populations where anti-psychotics are less commonly used indicate that many of these deficits are not an artifact of medications. However, not all studies of at-risk groups include only anti-psychotic naïve participants, and in patient groups, medications may be exacerbating these findings. Similarly, cognitive deficits in patients with schizophrenia may also be confounding these motor findings as motor performance is certainly closely linked to cognitive function [e.g., Ref. (109, 110)].

Finally, as noted throughout, and with the exception of motor learning, across most domains evidence directly linking these motor behaviors to the cerebellum are generally lacking. As such, the implication of cerebellar dysfunction is relatively indirect. By combining these motor measures with neuroimaging techniques we can better investigate the cerebellum in psychosis. Importantly, by combining these behavioral measures with measures of cerebellar volume or function, we can more effectively establish whether or not these behaviors may serve as markers of disease, and in clinical high-risk populations, they may serve as predictive biomarkers, as recently suggested by Bolbecker and colleagues (52). However, the cerebellum is a relatively large structure that is involved in both motor and non-motor behaviors (34, 35). An understanding of the cerebellar functional topography (4, 111) is important when considering the structure and its role in disease. It is important to consider the regional and functional organization within the cerebellum when looking to link the structure to overt motor deficits seen across the psychosis spectrum.

## Cerebellar Functional Topography

Beginning in the mid 1980s, investigators began speculating about the non-motor role of the cerebellum (28–31, 112). Investigations in non-human primates provided additional support for this notion. Using viral tract tracing methods, distinct tracts connecting pre-frontal and primary motor regions of the cortex to the cerebellum were revealed (113–116). These closed-loop circuits provide topographically segregated connections with the cerebral cortex. Specifically, the anterior aspects of the cerebellum (largely lobule V, but also lobules IV, and VI) along with a region in the inferior posterior cerebellum (lobules VIIIa and VIIIb) were connected to the primary motor cortex. Conversely, lateral aspects of the cerebellum (Crus II) were connected to the pre-frontal cortex (113). Similar motor and pre-frontal dissociations were seen in the dorsal and ventral aspects, respectively, of the cerebellar dentate nucleus (115).

**Figure 1:**
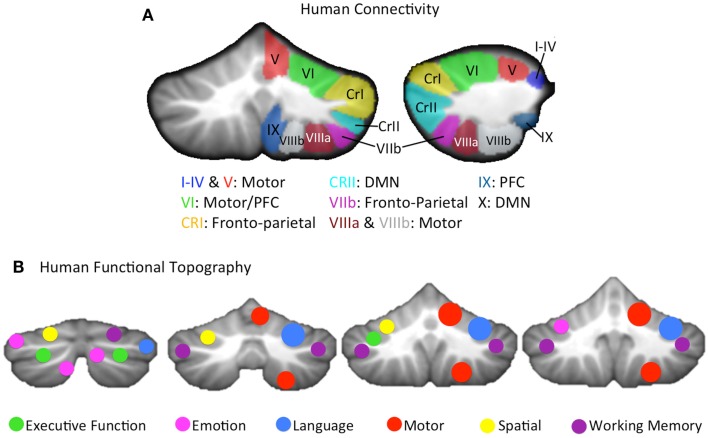
**(A)** A lobular summary of resting state connectivity (left: coronal, right: sagittal) analyses conducted in humans, shown for the right cerebellar hemisphere, and concatenated across several studies (105, 121). Lobules of the right hemisphere are labeled, and general connectivity patterns are listed. The cerebellar vermis, made up of mid-line lobular aspects analogous to the hemispheres was not included, but distinct connectivity patterns, comparable to those seen in their hemispheric counterparts, have been reported (105). **(B)** A summary of the cerebellar functional topography presented on coronal slices (left to right, posterior to anterior), as demonstrated in humans using meta-analysis (4, 123) as well as functional neuroimaging (111) is provided. Verbal and spatial processing is differentially lateralized, and motor and non-motor processing patterns are relatively consistent with cerebellar sub-regions that are associated with motor and non-motor cortical regions, respectively. Motor activation was largely localized to the right hemisphere, given that only right handed individuals were included in these investigations. CRI, Crus I; CRII, Crus II; DMN, default mode network; PFC, pre-frontal cortex.

More recently, using both structural (DTI) and functional connectivity neuroimaging (fcMRI) methods, a parallel topography of connections has been demonstrated in the human brain as well. fcMRI has revealed comparable distinct motor and cognitive networks in the cerebellar hemispheres at rest, based on the correlation between the resting state brain signal in these regions (117–119), and the dorsal and ventral dentate distinction was also replicated in humans using this methodology (120). However, distinct cerebello-cortical networks go beyond just a general motor/pre-frontal (non-motor) distinction. By investigating the resting state cerebello-cortical networks of individual cerebellar lobules, Bernard and colleagues showed that on a lobular level, the cerebellum is uniquely coupled with distinct cortical regions, resulting in distinct networks (105). Further support for multiple distinct motor and non-motor cerebellar networks comes from the work of Buckner and colleagues (121) (Figure 1A). They created a cerebellar parcelation based on coupling with multiple cortical resting state networks of the cortex. Finally, DTI has demonstrated distinct white matter tracts connecting the cerebellum and the cortex, that nicely parallel non-human primate literature (122). Thus, the cerebellum has distinct motor and non-motor closed-loop circuits with the cerebral cortex.

While the dissociable structural and functional motor and non-motor connections between the cerebellum and the cerebral cortex provide evidence for a topographically distinct functional organization in the cerebellum, there is also additional evidence from lesion studies and functional neuroimaging. The notion of a cognitive affective syndrome due to damage in the posterior lobe of the cerebellum was initially described by Schmahmann and Sherman (31). More recently, several investigations by Stoodley and colleagues (4, 111, 124) using both meta-analysis and functional neuroimaging indicate that in addition to the closed-loop circuitry of the cerebellum, there is also a distinct topography of functional activation across different task domains (Figure 1B). That is, tasks such as working memory and motor tapping result in activations in distinct cerebellar regions. Likewise, language and spatial processing are lateralized as in the cortex (4, 111), and there is some evidence of a unique area active when processing emotion (4). Even within the anterior cerebellum, there appear to be sub-regions associated with distinct types of motor behavior and learning (88). Finally, in healthy individuals, regional cerebellar volume is also associated with individual differences in behavioral performance on motor and cognitive tasks in regionally specific ways (58). Importantly, there is increasing evidence to indicate that the cerebellum is processing non-motor information in many of these higher order cognitive tasks (125), and that cerebellar contributions are necessary for effective task performance, and not just an artifact of the structure’s connections with the cerebral cortex (33). Given the diverse functional contributions of the cerebellum and the topographical nature of the activation associated with these task domains, the potential benefits of more targeted regional investigations are clear. Distinct cortico-cerebellar circuits and cerebellar regions may be differentially impacted in the disease state, and consideration of this putative regional variability may provide additional clarity for our understanding of the cerebellum in psychosis. Not only might this provide key insights into the motor deficits seen in schizophrenia and psychosis-risk, but it may also shed light on the various cognitive deficits that accompany the disorder.

## Regional Cerebellar Investigations

Converging evidence indicates that there are distinct functional sub-regions within the cerebellum making up a functional topography within the structure. Furthermore, the sub-regions of the cerebellum are part of distinct motor and non-motor cortical circuits. As such, when investigating the cerebellum it is important to consider the structure regionally. Making up approximately 10% of the total brain volume, the cerebellum is especially large to be considered as a whole. Its size, coupled with the known functional sub-regions call for a more fine-grained approach. It may be that sub-regions of the cerebellum are differentially impacted in the disease state. Not only would this approach provide important insight into the cerebellum across the psychosis spectrum, but it would also allow for comparisons across psychopathology. Cerebellar morphological differences with respect to controls have been observed in both depression and bipolar disorder (126–129). Understanding whether or not individual sub-regions or lobules are impacted differently across disease types will increase our understanding of the cerebellar contributions to psychopathology.

Post-mortem investigations provided some of the first evidence to indicate that there are cerebellar morphological differences in patients with schizophrenia with respect to controls. Differences when compared to controls have indicated reduced gyrification in the cerebellar vermis in patients (130), decreased neuronal integrity in patients, also in the cerebellar vermis (131), and there is a decrease in Purkinje cell (one of the main cerebellar cell types) density (fewer Purkinje cells) in patients with schizophrenia, as compared to controls (132). However, these results have been somewhat mixed with some groups showing no differences in patients relative to controls (133–135). These mixed findings may be because cerebellar deficits are specific to certain sub-types of schizophrenia as suggested by Lohr and colleagues (133), though regional sampling (or lack thereof) may also come into play in these investigations. Despite the mixed results, this work has provided important insights into the cerebellum in schizophrenia, particularly with respect to the underlying cytoarchitectonic pathology. That is, any possible volumetric differences seen using neuroimaging may be due to the decreased neuronal integrity and smaller number of Purkinje cells revealed in these post-mortem investigations. However, one weakness in this method is the fact that in at-risk populations (both genetic and UHR), there is a lack of post-mortem data as these individuals are typically quite young. Thus, we can only make inferences regarding cerebellar cytoarchitecture in these populations, and are reliant upon neuroimaging research.

Thanks to *in vivo* neuroimaging methods, there is increasing evidence to indicate that there are morphological differences in the cerebella of patients with schizophrenia, and there is an emerging literature indicating this in psychosis-risk as well (both UHR and genetic). However, the results thus far are relatively mixed [for a review, see Ref. (28)]. That is, in some cases, patients with schizophrenia have larger cerebellar volumes than controls, whereas in other cases, cerebellar volume in patients is decreased with respect to controls [cf. (28)]. Subsequent to the review of Shenton and colleagues, additional mixed findings with respect to cerebellar volume in schizophrenia have been revealed (136–142). The majority of these studies were methodologically similar in that they looked at the cerebellar hemispheres as a whole, and also investigated the vermis, which was often further subdivided into vermal sub-regions. The most consistent differences were found in the vermis across studies (136, 140), and the more detailed approach taken to investigating the vermis may be a contributing factor. The literature on the cerebellum in those at-risk for psychosis (UHR and genetic risk) is much smaller than that in schizophrenia, but the mixed findings persist (2, 142–144). However, they did differ methodologically, largely relying upon whole brain methods to assess gray matter. While this is not an exhaustive list of investigations of the cerebellum in schizophrenia and psychosis-risk, it is clear that the results are mixed, and while differences in study inclusion factors and subject age may contribute, we suggest that the gross measures of cerebellar volume, particularly in the hemispheres are a contributing factor.

Assessments of the cerebellum taking regional approaches are certainly the exception to the rule, and the few cases where these approaches have been used have provided interesting results. In a relatively small sample (*n* = 19), Loeher and colleagues (141) traced individual cerebellar lobules and found volumetric differences in the vermis. In childhood-onset schizophrenia, siblings, and healthy controls, Greenstein and colleagues also investigated cerebellar sub-regions (142). Though they did not look at individual lobules, they did subdivide the cerebellar hemispheres based on anatomical boundaries, providing increased detail relative to whole hemisphere analyses. In this study, the patients with schizophrenia (*n* = 94) had smaller anterior cerebellar volume, as well as smaller vermis volume when compared to controls, and they also showed differing developmental volumetric trajectories with respect to controls. Though the siblings of the patients did not differ in their regional cerebellar volumes from controls at baseline, they did show differing regional volumetric trajectories during longitudinal assessments (142). The detailed approaches taken here, along with the large sample size provide important insights with respect to cerebellar volume across the schizophrenia spectrum, and indicate that cerebellar sub-regions may be differentially impacted. As discussed above, Edwards and colleagues (43) investigated the anterior and posterior cerebellum with respect to eye-blink conditioning. They found smaller anterior cerebellar volume in patients with schizophrenia, and though there were no significant correlations with behavior in the patient group, the relationships were in the opposite direction as compared to controls. The authors as a result suggested that there may be altered structure–function relationships in schizophrenia with respect to the cerebellum and eye-blink conditioning (43). Using automatic lobular segmentation methods (58) we recently demonstrated that there are regional lobular differences in UHR adolescents and young adults (2). The anterior cerebellum and Crus I differed between the patient group and age-matched healthy controls, as did the vermis, though lobule X did not differ. Though this study was focused on specific cerebellar lobules, this provides further preliminary evidence that regional cerebellar volumetric differences may be present prior to the development of psychosis, and as we continue to longitudinally investigate these individuals, we will be able to investigate their volumetric trajectories over time. Finally, an intriguing new study looking at modularity of the cerebellum using DTI, indicates that the modular organization of the cerebellum is altered in schizophrenia (145). This finding further underscores the importance of regional approaches, as the functional architecture of the cerebellum seems to differ in schizophrenia (93), and these structural findings may underlie this (145).

It is clear that cerebellar-mediated motor behaviors are impacted in patients with schizophrenia, and across the psychosis spectrum. There are also cognitive deficits that may be linked, at least in part, to the cerebellum. From a morphological or network perspective, the contributions of the cerebellum are better understood by investigating this structure regionally. This may provide key insights into both the motor and cognitive deficits experienced by patients with schizophrenia and psychosis-risk groups. In the work summarized above, though the cerebellum is implicated in these motor deficits, direct links between morphology and performance are generally lacking. By taking a regional approach, specific hypotheses with respect to the cerebellum and motor performance can be defined and tested to better understand cerebellar-motor deficits across the psychosis spectrum. Applying this approach to procedural learning in UHR populations, we demonstrated that volume of Crus I in at-risk individuals was positively correlated with procedural learning (2). Interestingly, this motor task was associated with a more cognitive region of the cerebellum, suggesting that cognitive circuits, which are implicated in complex motor tasks (106) are perhaps also implicated in the cerebellar-motor deficits seen across the psychosis spectrum. Future investigations including regional measures of cerebellar volume with respect to motor deficits are warranted, and will likely provide important insights into the involvement of this structure in the disease state.

Most importantly, it is now much easier to investigate the cerebellum, especially morphology, on a lobular basis. While in the past such detailed analyses would require precise hand tracing of individual cerebellar lobules requiring large amounts of time and multiple raters, several recent studies have presented automatic lobular segmentation methods (58, 146, 147). These methods allow for investigators to easily compute lobular cerebellar volumes, and can be applied to large clinical samples, eliminating much of the methodological challenge associated with hand tracing. Bernard and Seidler (58) used the lobular delineations and masks originally created by Diedrichsen and colleagues as part of the SUIT atlas (148, 149). We successfully applied these methods in an investigation of adolescents and healthy controls at ultra-high risk for psychosis (2), demonstrating their utility in clinical populations, and providing important information about both regional cerebellar volume as well as motor learning in this population. Similarly, the lobular delineations available in the SUIT atlas can be used as starting seed regions for resting state connectivity analyses (1, 105, 150), and such analyses have revealed interesting differences and associations between lobular cerebellar connectivity, postural control, and symptom severity in UHR individuals (1). Finally, these regions may also serve as useful starting points for DTI analyses. Salmi and colleagues looked at cerebello-thalamo-cortical white matter networks in healthy adults (122), and similar analyses across the psychosis spectrum would be beneficial to our understanding of the role of the cerebellum in motor deficits, as well with respect to the disease state more generally.

## Conclusion

A range of cerebellar-mediated motor tasks are impacted across the psychosis spectrum. Such motor impairments are present prior to disease onset, and may serve as a marker for pre-morbid cerebellar dysfunction. However, to date, direct links between these motor impairments and cerebellar morphology and/or cerebello-cortical networks have generally been lacking. In part, this may be due to standard approaches that treat the cerebellum as a functionally homogenous brain structure, though converging evidence indicates that there are distinct motor and non-motor functional regions within the cerebellum [e.g., Ref. (16, 23, 34, 90)]. Thus, we suggest that more specific topographically informed approaches to investigating the cerebellum (particularly with respect to cerebellar morphology and cerebello-cortical networks) across the psychosis spectrum will yield informative results with respect to the involvement of the cerebellum in psychosis. Such analyses will provide important information with respect to motor dysfunction, and they also may shed light on mixed findings with respect to cerebellar morphology. Furthermore, such an approach may provide additional insights into cognitive deficits experienced by these patient groups. That is, regional volume or functional differences may be limited to sub-regions of the cerebellum, and investigations of the structure as a whole may have masked these important findings. A better understanding of regional cerebellar morphological and functional differences will indicate whether or not there are more global or local cerebellar deficits in psychosis. Cerebellar-mediated movement abnormalities may be an overt manifestation of a more general cerebellar deficit, and regional analyses will allow this to be tested more directly. However, it is of note that these insights will be on a macro-structural level and potential underlying cellular differences and cellular contributions to pathophysiology are not assessed with these volumetric techniques. Computational models with respect of cerebellar cytoarchitectonics may be especially informative in that domain. Finally, regional approaches to investigating the cerebellum and cerebellar-motor abnormalities across disorders will allow for important comparisons resulting in a better understanding of the cerebellum across disorders.

## Conflict of Interest Statement

The authors declare that the research was conducted in the absence of any commercial or financial relationships that could be construed as a potential conflict of interest.
